# Application of perovskite type oxide La_*x*_Ce_1−*x*_coo_3_ in lignin depolymerization and mono-phenol preparation

**DOI:** 10.1039/d4ra07918c

**Published:** 2025-01-17

**Authors:** Tao Zhu, Yujie Wang, Miao Chang, Jie Xu, Xiaobin Hao

**Affiliations:** a School of Materials and Chemical Engineering, Chuzhou University Chuzhou Anhui 239000 China wangyj120@163.com; b School of Life Sciences, Anhui University Hefei Anhui 230601 China

## Abstract

This study successfully prepared La_*x*_Ce_1−*x*_CoO_3_ (*x* = 0.2, 0.4, 0.6, 0.8, 1) series perovskite oxide catalysts by co precipitation, and found that La_0.6_Ce_0.4_CoO_3_ can significantly improve the yield of bio-oil under specific conditions (lignin to catalyst mass ratio of 1 : 2, reaction temperature of 240 °C, time of 10 hours, using methanol and ethanol as solvents). Furthermore, NH_3_-TPD, GC-MS, and FTIR analyses revealed the thermal decomposition behavior of key bonding structures such as β-O-4 and C–O–C during the catalytic process, while generating various mono-phenolic products such as guaiacol and 2,6-dimethylphenol. In addition, studies have shown that the physicochemical properties of perovskite type oxide catalysts have a significant impact on the chemical properties of lignin oil. By increasing the acidity of the catalyst, not only can the yield of lignin oil and phenols be improved, but also the yield of carbon can be reduced. More importantly, the catalyst performed well in the test of lignin catalyzed hydrogenation to produce monophenols, significantly improving the conversion rate of lignin and the yield of various monophenols compared to noncatalytic processes.

## Abstract 2

Possible pathways of SLS catalytic depolymerization to monomeric phenols.

## Graphical abstract

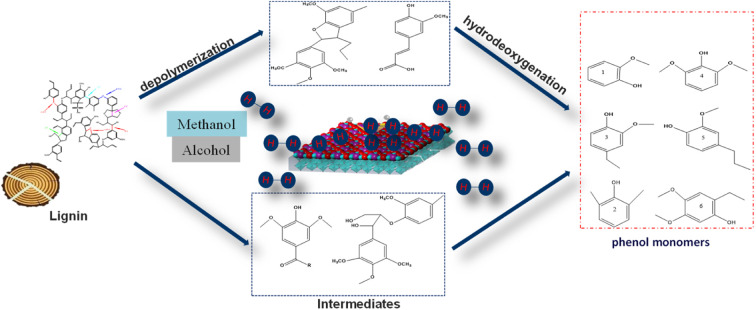

## 1 Introduction

With the increasingly severe global energy crisis and environmental issues, developing efficient and environmentally friendly energy conversion and storage technologies has become an important topic in today's scientific research.^1^ Among numerous renewable biomass resources, lignin is the only nonpetroleum resource in nature that can provide renewable aromatic compounds. Its efficient utilization is of great significance for promoting the development of green chemistry and bioenergy.^2^ Lignin is a three-dimensional amorphous high molecular weight aromatic polymer mainly composed of three phenylpropane structural units, namely hydroxyphenyl, guaiacyl, and syringayl, connected by C–O–C and C–C bonds.^3,4^ However, due to the complex structure, strong stability, and large molecular size of lignin, its effective utilization has always been a challenge.

In order to fully utilize lignin and make it profitable, we need to rely on depolymerization technology to extract the platform chemicals from lignin. At present, various thermochemical methods have been studied, which can depolymerize lignin into monomers and dimers, mainly alkaline hydrolysis,^5^ acid hydrolysis, pyrolysis,^6^ oxidation,^7^ and hydrogenolysis.^8^ Among them, hydrogenolysis is one of the effective methods for depolymerizing lignin into phenolic biooil, and the reaction conditions are mild. For hydrogenolysis, the source of hydrogen can be H_2_ molecules or hydrogen donating reagents. It is worth noting that in the process of lignin hydrolysis, alcohol solvents, especially methanol, ethanol, and isopropanol, are widely used due to their unique properties. These solvents exhibit high efficiency in dissolving lignin and possess potential hydrogen donor characteristics, making them particularly outstanding solvent choices in lignin hydrolysis processes.^9^ Barta *et al*.^10^ used supercritical methanol as a solvent and Cu doped porous metal oxides as catalysts, effectively promoting the depolymerization of lignin and converting it into high-value monomer compounds. Ma *et al*.^11^ used isopropanol as a solvent and hydrogen transfer promoter, and employed Ni/ZrP catalyst to hydrogenate lignin. The results showed that isopropanol was more effective in H_2_ generation and facilitated the hydrogenation deoxygenation of lignin derived phenolic compounds, inhibiting the re polymerization reaction between unstable intermediates and phenols.

However, the hydrogenation of lignin into high yield, high selectivity, and low-cost monophenols remains a challenge. To address these issues, various catalysts have been used to break the ether bonds of lignin, including homogeneous catalysts and heterogeneous metal catalysts. Metal heterogeneous catalysts have the advantages of selectivity, reusability, and no emission of pollutants, and are widely used in the catalytic cracking of lignin and lignin model compounds. Metal catalysts are generally dominated by precious metals such as ruthenium,^12^ platinum,^13^ palladium, *etc.* Although precious metals can provide many attractive properties, they are difficult to meet the needs of industrial scale due to their high price and limited quantity. Therefore, it is necessary to develop inexpensive catalysts with better catalytic effects. Some inexpensive metals, such as Ni,^14^ Fe,^15^ Cu,^16^ Co,^17^*etc.*, have been explored in some research reports.

Zhao *et al.*^18^ achieved selective hydrogenolysis of renewable lignin using low-cost catalyst Ni/MgO, achieving a lignin conversion rate of 93.4% and a phenolic monomer yield of 15.0% under optimal reaction conditions. Compared with pure MgO, the introduction of Ni can effectively improve the conversion rate of lignin and increase the yield of bio-oil. Co based catalysts also exhibit good performance in catalyzing hydrogenolysis. Recently, some researchers have turned to bimetallic catalysts because compared to single metal catalysts, bimetallic catalysts can form more catalytic centers through interactions between metal atoms. These catalytic centers can more effectively bind with reactants in the reaction, promoting the progress of the reaction.

Jiang *et al.*^19^ investigated the catalytic performance of Ni Pd bimetallic catalysts for lignin depolymerization. Among them, the Ni50Pd50/SBA-15 catalyst has the best depolymerization effect on corn stover lignin, with the highest total mono-phenolic yield (8.14%), which is 1.96 times that of the single metal catalyst Ni/SBA-15 and 1.44 times that of Pd/SBA-15. Therefore, developing an inexpensive bimetallic catalyst to catalyze the depolymerization of lignin is of great significance.

Perovskite materials, with their unique crystal structure and excellent physical and chemical properties, have demonstrated extraordinary application potential in many fields. In the field of energy conversion,^20,21^ perovskite materials have become a key component in systems such as solar cells and fuel cells due to their efficient energy conversion capabilities, playing a crucial role and greatly promoting the development and effective utilization of clean energy. In the field of materials science, the tunable optoelectronic properties and stability of perovskite materials make them ideal candidates for photodetectors and storage devices.^22,23^ In addition, in the field of catalysis, perovskite materials are widely used in catalytic oxidation,^24,25^ photocatalytic water splitting,^26^ and environmental pollutant degradation^27^ due to their abundant active sites and good stability, demonstrating excellent performance.

Lignin depolymerization, as a key step in biomass conversion, also requires catalytic materials with high activity and stability to break its complex molecular structure and achieve high selectivity and efficiency in conversion. The performance characteristics exhibited by perovskite materials in other fields, such as controllable active sites, high stability, and good adaptability to complex chemical reactions, are highly compatible with the core requirements of lignin depolymerization process.

Especially, the ability of perovskite materials to optimize catalytic performance by finely regulating their composition and structure makes them an ideal catalyst for lignin depolymerization. Therefore, inspired by the achievements of perovskite materials, introducing them into the field of lignin depolymerization for research is not only a natural extension of their application, but also an important exploration direction for efficient utilization of biomass resources. This cross disciplinary attempt will help reveal the potential of perovskite materials in biomass catalytic conversion, promoting the efficient conversion of biomass into high value-added chemicals and energy.

Among them, La_*x*_Ce_1−*x*_CoO_3_, as a new type of perovskite catalyst, not only inherits the high stability characteristics of traditional perovskite materials, but also achieves catalytic performance control by regulating the composition of A-site ions (such as La/Ce ratio), providing the possibility for efficient implementation of specific catalytic reactions. This unique tunability has increasingly attracted attention to the application research of La_*x*_Ce_1−*x*_CoO_3_ in fields such as catalytic oxidation and photocatalysis, and has achieved a series of remarkable results. However, despite the enormous potential of La_*x*_Ce_1−*x*_CoO_3_ in various catalytic fields, its research in the key biomass conversion field of lignin depolymerization is almost blank.

Lignin, as a renewable resource with abundant reserves in nature, its effective depolymerization and transformation are of great significance for the development of sustainable energy and chemicals. Given this, we have reason to believe that by preparing La_*x*_Ce_1−*x*_CoO_3_ catalysts with different La/Ce ratios and systematically studying their performance in catalyzing the depolymerization of sodium lignosulfonate, we aim to reveal the relationship between catalyst structure and performance, and provide new approaches and strategies for the efficient conversion and utilization of biomass resources. Specifically, this study will comprehensively evaluate the catalytic activity, selectivity, and stability of La_*x*_Ce_1−*x*_CoO_3_ catalyst in the sodium lignosulfonate depolymerization reaction through steps such as catalyst preparation, characterization, and performance testing. At the same time, combined with the structural analysis of catalysts, key scientific issues such as active sites and reaction mechanisms of catalysts are explored in depth, providing scientific basis for further optimizing catalyst performance and expanding its application scope. In summary, this study not only has significant academic value, but also has important practical significance for promoting the high-value utilization of lignin and promoting the sustainable development of biomass resources.

## 2 Experimental section

### 2.1 Catalyst preparation

La_*x*_Ce_1−*x*_CoO_3_ (*x* = 0.2, 0.4, 0.6, 0.8, 1) was prepared by co precipitation method, and a transparent solution was prepared by mixing stoichiometric amounts of lanthanum nitrate hexahydrate, cerium nitrate hexahydrate, and cobalt nitrate hexahydrate. Subsequently, under stirring in an 80 °C water bath, a precipitant sodium carbonate solution was added to adjust the pH value to 8.0–8.5, resulting in a purple flocculent mixture. Then precipitate the mixture overnight and filter under vacuum. The sample obtained by filtration was dried in a drying oven at 110 °C for 12 hours, and then calcined in a muffle furnace at 800 °C for 5 hours to obtain La_*x*_Ce_1−*x*_CoO_3_ (*x* = 0.2, 0.4, 0.6, 0.8, 1) perovskite samples.

### 2.2 Catalyst characterization

The BSD-PS2 analyzer was used to measure the N_2_ adsorption desorption isotherm at N_2_ isothermal (196 °C), and the pore structure and specific surface area of the catalyst were determined. Calculate the specific surface area, pore size, and pore volume using BET equation and BJH model, respectively. The crystal phase analysis of the sample was carried out using a powder X-ray diffractometer (XRD) equipped with Cu Kα radiation (1¼ 0.154 nm), 40 kV, 30 mA, and a scanning range of 10–80. In order to observe the morphology and dispersion of surface elements, scanning electron microscopy (SEM) analysis and energy dispersive X-ray spectroscopy (EDS) plotting were used. The elemental states of metals were measured using X-ray photoelectron spectroscopy (XPS) experiments on a Thermo-Fisher Scientific K-A apparatus. The acidity of the catalyst was measured using a BELCAT-B instrument from Osaka, Japan, and the acidic sites under NH_3_ gas were determined using TCD.

### 2.3 Depolymerization of lignin

The depolymerization experiment of lignin was conducted in a high-pressure reactor. Firstly, add 0.6 g of sodium lignosulfonate and 0.3 g of catalyst (with a mass ratio of lignin to catalyst of 2 : 1) into the reaction vessel. Then add 10 mL of isopropanol and 10 mL of ethanol. Finally, heat the reactor to the expected temperature (180–260 °C) and react for 6–14 hours. After the reaction is complete, cool the reactor to ambient temperature. The reaction product is divided into three parts: gas, liquid, and solid. Due to the relatively small gas production rate (less than 0.5 wt%), it can be ignored. Use filtration method for solid–liquid separation and wash the kettle body with anhydrous ethanol. The obtained liquid product is subjected to solvent removal under a rotary evaporator to obtain biooil. In order to recycle the used catalyst, the coke, catalyst, and unreacted lignin were washed with acetone and dimethyl sulfoxide solution, respectively. The mixed residual solid obtained after washing was dried and ground into powder, and then calcined in a muffle furnace. The obtained solid is the regenerated catalyst. Repeat all experiments three times and take the average to reduce errors.

### 2.4 Analysis of lignin conversion products

Gas chromatography-mass spectrometry (GC-MS, Agilent 7890A) and HP-5MS capillary column (30 m × 0.25 mm × 0.25 μm) were used to detect and identify the components of bio-oil. The working conditions of GC-MS are: the initial temperature of the column temperature chamber is 80 °C, and then the temperature is raised to 200 °C at a rate of 10 °C min^−1^. The quantitative analysis of phenolic monomers after the reaction and the solvent analysis collected after the reaction were performed using flame ionization detection gas chromatography (GC-FID, Agilent 6890N). The chromatographic column is an HP-5 MS capillary column (30 m × 0.25 mm × 0.25 μm). The working conditions of GC-FID are as follows: maintain the column temperature at 70 °C for 2 minutes, then increase it to 200 °C at a rate of 10 °C min^−1^, and maintain it for another 2 minutes. The yields of biooil, monomers, and charcoal are determined by the following formula:
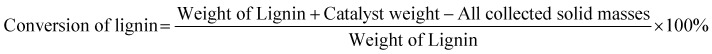

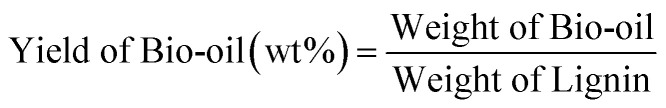

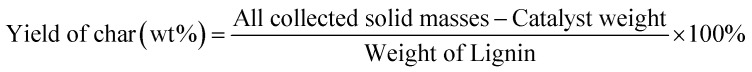

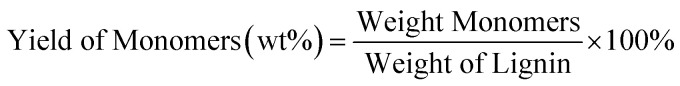


The changes in functional groups of lignin and product biooil were detected using the VERTEX 80V infrared spectroscopy (FT-IR) instrument from Bruker, Germany. Solid KBr was used for compression, with a sample to KBr mass ratio of 1/100 and a detection wavelength range of 4000–400 cm^−1^. Each spectrum was scanned 32 times.

Determine the elemental composition content of raw lignin and product biooil using 2400 II PE from the United States. Wrap the test sample in aluminum foil, set the O_2_ and He flow rates to 30 mL min^−1^ and 250 mL min^−1^, respectively, and burn at 1200 °C. The content of C, H, and N elements can be measured, and the content of O element can be obtained using the law of conservation of mass. And the Dulong formula can be used to calculate the high calorific value of related products: HHV (MJ/Kg) = 0.335 × C + 1.422 × H − 0.154 × O − 0.145 × N.

## 3 Results and discussion

### 3.1 Characterization of La_*x*_Ce_1−*x*_CoO_3_ catalysts

#### 3.1.1 XRD

**Fig. 1:**
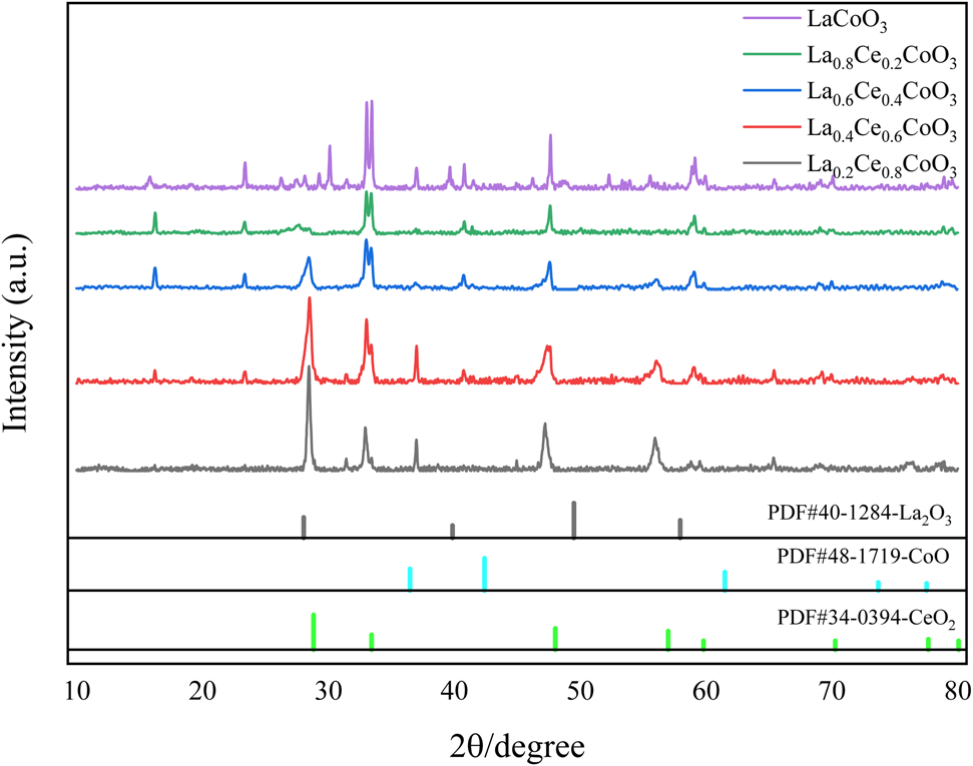
XRD spectra of La_*x*_Ce_1−*x*_CoO_3_ (*x* = 0, 0.2, 0.4, 0.6, 0.8) series catalysts.

The crystal structure of La_*x*_Ce_1−*x*_ CoO_3_ (*x* = 0.2, 0.4, 0.6, 0.8, 1) perovskite catalysts was analyzed by X-ray diffraction, and the results are shown in Fig. 1. Observation revealed that all catalyst samples exhibited typical perovskite diffraction peaks. As the content of Ce component increases, the width of XRD peaks gradually decreases and the peak shape becomes sharper. Especially around 2*θ* of 28.5°, 32.9° and 47.5°, clear diffraction characteristic peaks appeared, which were analyzed by MDI Jade6.0 software and consistent with the standard data of CeO_2_ (JCPDS NO. 34-0393). It is particularly noteworthy that when the doping amount of Ce reaches 0.6 and 0.8, no obvious impurity peaks are observed in the XRD curve, and the intensity of the formed oxide diffraction peaks is high. This indicates that the doping of Ce did not cause significant lattice distortion, and the generated perovskite is still in the pure phase.

The occurrence of this phenomenon may be due to the following reasons: in traditional perovskite structures, A-site ions are usually divalent or trivalent, while Ce^3+^ ions are more unstable and easily oxidized to Ce^4+^ ions. Therefore, in the catalyst, Ce mainly exists in the state of Ce^4+^. However, if all Ce^4+^ ions enter the structure of perovskite, it will lead to charge imbalance in the La_*x*_Ce_1−*x*_CoO_3_ catalyst. In order to compensate for this charge imbalance, some La and Ce ions did not participate in the formation of the perovskite structure, resulting in a corresponding number of B-site transition metal ions not entering the perovskite structure, but existing in the form of their respective oxides.

#### 3.1.2 SEM-EDX

**Fig. 2:**
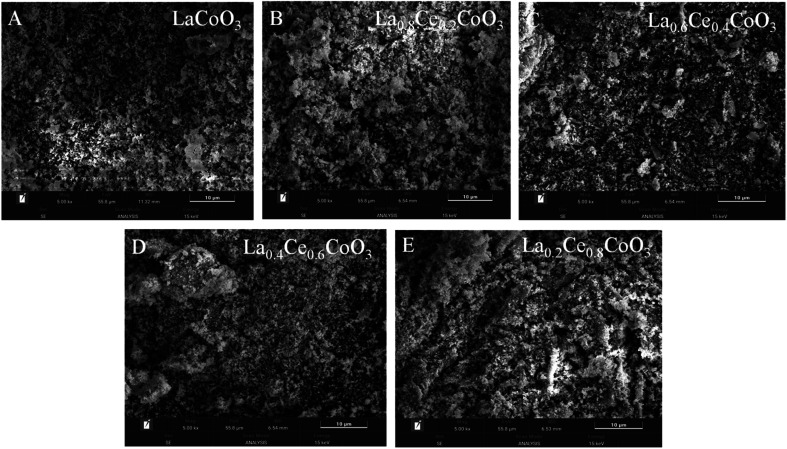
SEM image of La_*x*_Ce_1−*x*_ CoO_3_ catalysts ((A) *x* = 0, (B) *x* = 0.2, (C) *x* = 0.4, (D) *x* = 0.6, (E) *x* = 0.8).

The microstructure of La_*x*_Ce_1−*x*_CoO_3_ (*x* = 0, 0.2, 0.4, 0.6, 0.8) series perovskite catalysts was characterized and analyzed using scanning electron microscopy (SEM). As shown in Fig. 2, all catalysts exhibit a granular structure, and there is no significant difference in the size of nanoparticles among the catalysts. From (B) to (E) in Fig. 2, it can be observed that as the Ce element content gradually increases, La element is greatly replaced by Ce element, which leads to a decrease in the high porosity of perovskite and affects the overall morphology of the catalyst. The originally smooth or relatively uniform surface of perovskite began to show a small amount of white particle aggregation. These newly formed white particles tightly cover the surface of the original perovskite particles, forming a new surface coating layer.

**Fig. 3:**
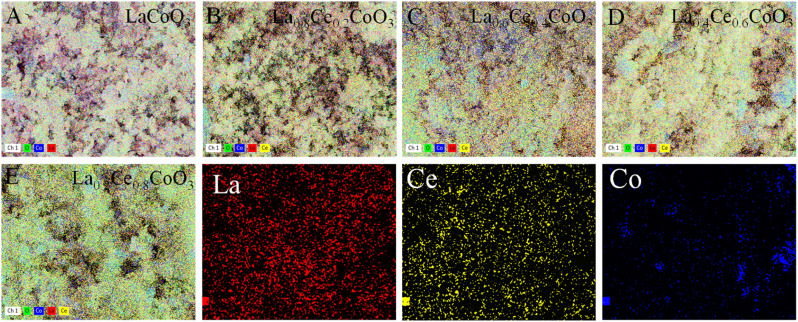
EDS spectrum of La_*x*_Ce_1−*x*_ CoO_3_ ((A) *x* = 0, (B) *x* = 0.2, (C) *x* = 0.4, (D) *x* = 0.6, (E) *x* = 0.8).

From the distribution of La element in Fig. 3, it can be seen that as the Ce content gradually increases, the concentration of La element gradually decreases, which is consistent with the expected design of La being partially replaced by Ce, further confirming the successful doping of Ce into the catalyst lattice. Specifically, in the La_0.6_Ce_0.4_CoO_3_ catalyst (Fig. 3C), La, Ce, O, and Co elements exhibit uniform distribution, indicating that all elements have good dispersibility within the catalyst. This good dispersibility may be a key factor in the superior catalytic performance of the La_0.6_Ce_0.4_CoO_3_ catalyst. It is worth noting that as the content of Ce element increases (as shown in Fig. 3D and E), the surface of the catalyst becomes rougher, which is attributed to the possible induction of uneven grain growth and the formation of irregular clusters by the addition of Ce element. In catalysts with lower Ce content, such as La_0.6_Ce_0.4_CoO_3_ (Fig. 3C), the appropriate introduction of Ce not only helps to improve the redox performance of the catalyst and promote the generation of active oxygen species, but also maintains the stability of the lattice structure, thereby gradually improving the catalytic performance.

#### 3.1.3 BET

**Fig. 4:**
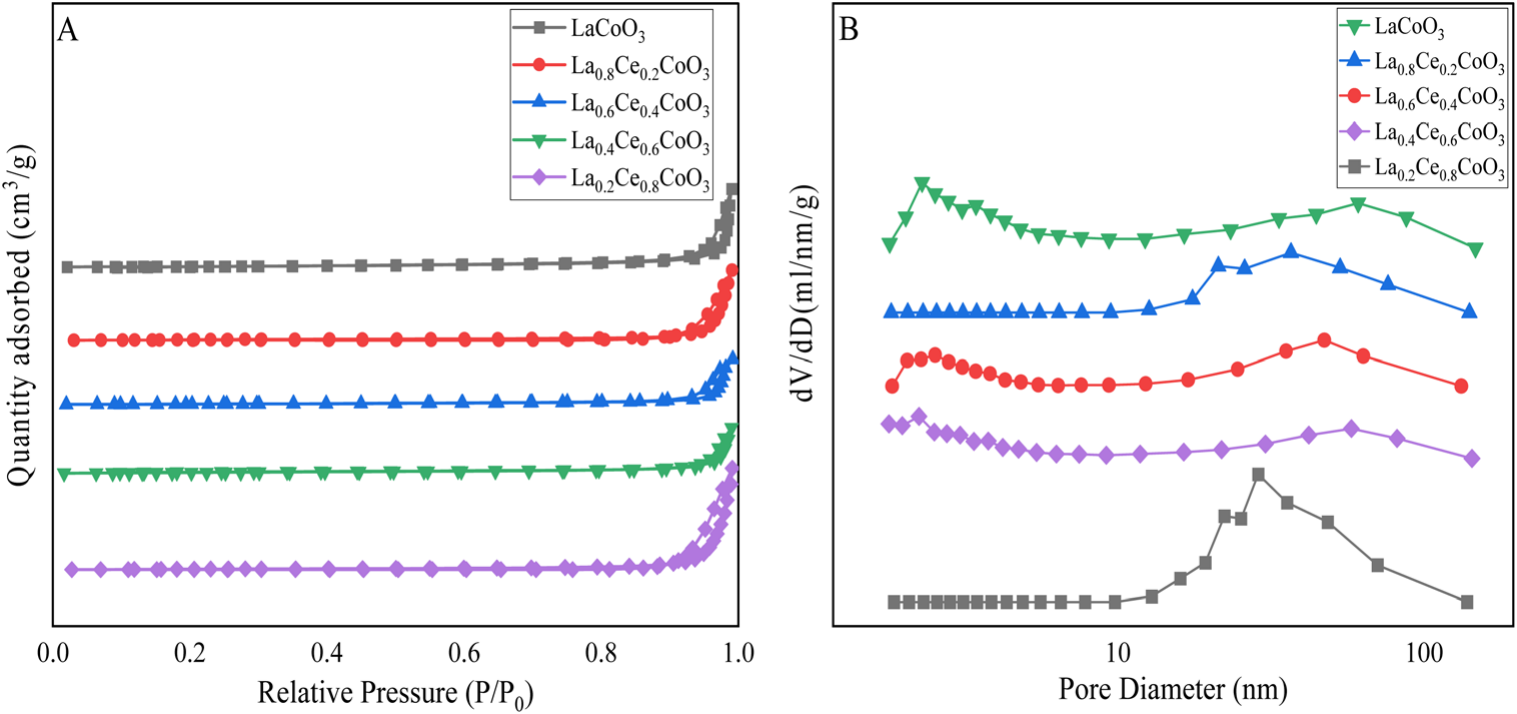
The nitrogen adsorption desorption isotherms (A) and pore size distribution curves (B) of La_*x*_Ce_1−*x*_CoO_3_ (*x* = 0, 0.2, 0.4, 0.6, 0.8) series catalysts prepared.

The N_2_ adsorption desorption isotherms of La_*x*_Ce_1−*x*_CoO_3_ series catalysts are relatively similar. According to the IUPAC classification standard, all prepared samples have type II adsorption isotherms, and H3 hysteresis loops appear in the range of relative pressure (*P*/*P*_0_) range of 0.7–1.0. For the La_*x*_Ce_1−*x*_CoO_3_ series catalysts, the isotherms in the part where *P*/*P*_0_ < 0.7 are linearly distributed, within this pressure range, N_2_ molecules undergo unrestricted single-layer or multi-layer adsorption on the catalyst surface. This adsorption mode is usually consistent with the characteristics of macro-porous materials, as macro-porous materials provide sufficient space for N_2_ molecules to freely diffuse and adsorb on the surface, when *P*/*P*_0_ > 0.7, a clear turning point appears on the isotherm, a typical H3 hysteresis loop. The appearance of this hysteresis loop is a clear indication of the existence of mesopores (Fig. 4).

In mesoporous materials, due to the small pore size, N_2_ molecules are subject to certain limitations during the adsorption process, resulting in incomplete reversibility of the adsorption and desorption processes, thus forming hysteresis loops on the isotherm.

Table 1 presents the specific surface area, pore size, and pore volume results of La_*x*_Ce_1−*x*_CoO_3_ (*x* = 0, 0.2, 0.4, 0.6, 0.8) perovskite catalysts. Among them, the specific surface area of the catalyst was calculated by the Brunner–Emmett–Teller (BET) method, while the pore size and pore volume were calculated by the Barrett–Joyner–Halenda (BJH) desorption method. According to the table, the specific surface area of La_*x*_Ce_1−*x*_CoO_3_ catalyst is significantly larger than that of LaCoO_3_ catalyst (6.2792 m^2^ g^−1^), indicating that cerium doping may alter the surface structure and active sites of LaCoO_3_. But when the doping ratio of Ce is 0.4, the specific surface area of the catalyst (4.1991 m^2^ g^−1^) is smaller than that of undoped (6.2792 m^2^ g^−1^), which may be due to the fact that doping sacrifices some of the specific surface area. Based on the XRD pattern, we can observe that although there were no significant impurity peaks observed at a Ce doping level of 0.4, the slight changes in lattice parameters indicate that the lattice structure has been finetuned, which may lead to lattice distortion or differences in ion radius, further affecting the surface structure of the catalyst. Secondly, the observation results of SEM images show that when the Ce doping amount is 0.4, the surface of the catalyst particles becomes smoother and the pore structure between particles is reduced. This morphological change is consistent with the specific surface area data calculated by BET method, that is, the specific surface area of La_*x*_Ce_1−*x*_CoO_3_ catalyst is smaller when Ce doping is 0.4 than when undoped. The phenomenon of surface smoothing and reduction of pore structure may be due to surface reconstruction caused by Ce doping. The surface reconstruction caused by doping may reduce the roughness and pore structure, thereby reducing the specific surface area. Although surface reconstruction may create new conditions for improving catalytic performance, it seems that this reconstruction has not yet reached its optimal state in La_0.6_Ce_0.4_CoO_3_ catalyst, resulting in a failure to maintain a high level of specific surface area.

**Table 1:** Surface structure properties of La_*x*_Ce_1−*x*_CoO_3_ (*x* = 0, 0.2, 0.4, 0.6, 0.8) series catalysts

Catalyst	Surface area (m^2^ g^−1^)	Pore volume (cm^3^ g^−1^)	Pore diameter (nm)
La_0.2_Ce_0.8_CoO_3_	6.9267	0.1349	77.9012
La_0.4_Ce_0.6_CoO_3_	6.5720	0.0690	41.9963
La_0.6_Ce_0.4_CoO_3_	4.1991	0.0671	63.9190
La_0.8_Ce_0.2_CoO_3_	8.7927	0.1024	46.5841
LaCoO_3_	6.2792	0.1186	75.5511

#### 3.1.4 XPS

**Fig. 5:**
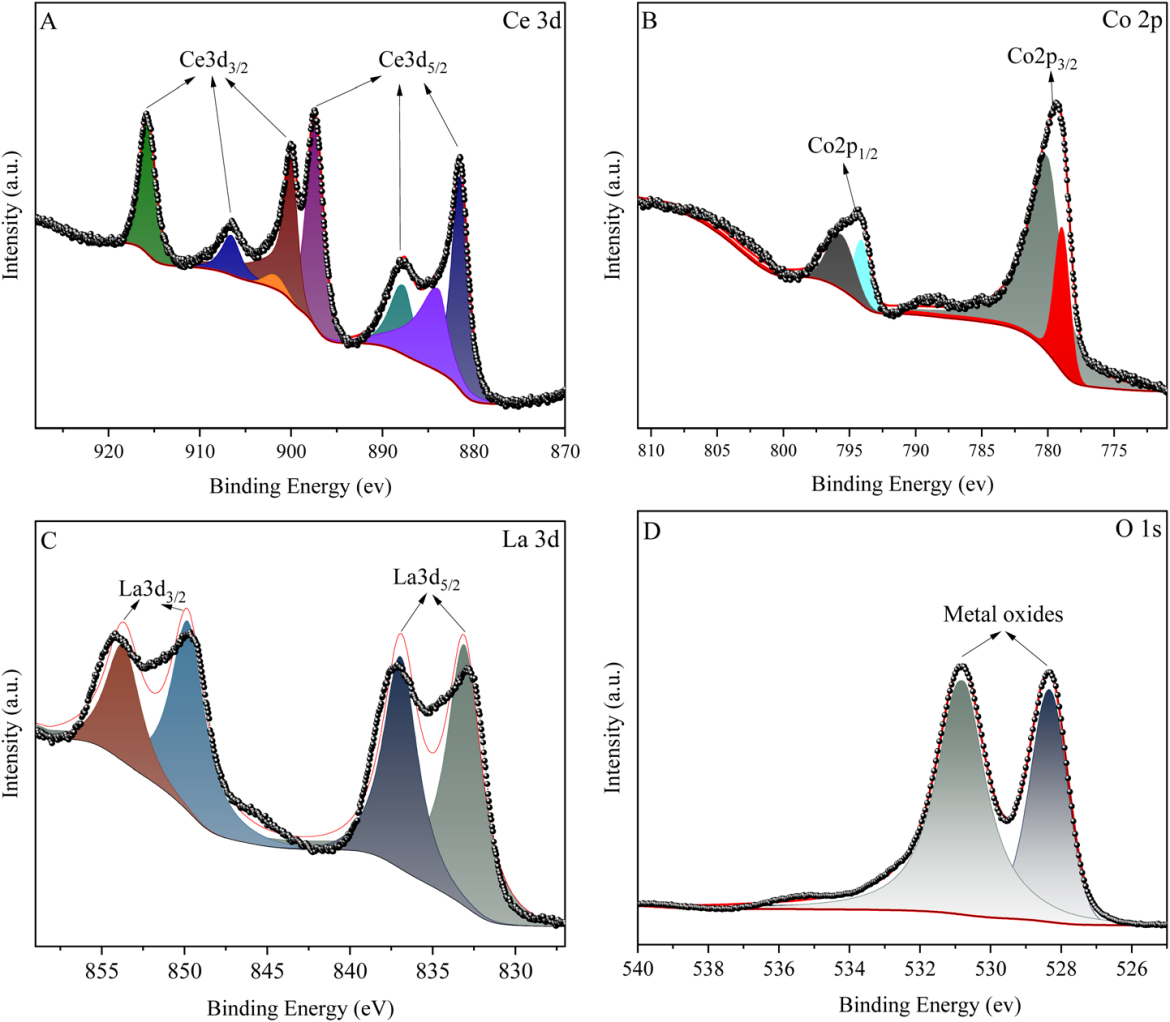
XPS spectrum of La_0.6_Ce_0.4_CoO_3_ catalyst. ((A) Ce 3d, (B) Co 2p, (C) La 3d, (D) O 1s).

Fig. 5 shows in detail the XPS spectrum and fitting curve of La_0.6_Ce_0.4_CoO_3_. From the figure, it can be clearly observed that the catalyst does indeed contain La, Co, Ce, and O elements, which is completely consistent with the chemical composition of the catalyst.

In the XPS spectrum of Ce_3d_ (Fig. 5a), eight peaks were successfully fitted. Among them, the four peaks of 899.99 eV, 901.77 eV, 906.58 eV, and 915.74 eV belong to the Ce_3d3/2_ region, while the four peaks of 881.51 eV, 883.88 eV, 887.69 eV, and 897.39 eV belong to the Ce_3d5/2_ region. Specifically, the peaks at 883.88 eV and 906.58 eV correspond to the characteristics of Ce^3+^, while the remaining peaks represent Ce^4+^ with different electronic configuration states. This indicates that in La_1−*x*_Ce_*x*_CoO_3_, the Ce element has a variable valence, but mainly exists in the form of Ce^4+^, which is attributed to the good thermal stability of Ce^4+^at higher calcination temperatures.

For the XPS spectrum of Co_2p_ (Fig. 5b), the Co_2p_ spectrum is divided into two asymmetric parts: Co_2p1/2_ and Co_2p3/2_. In the Co_2p3/2_ region, peaks with binding energies of approximately 778.92 eV and 780.06 eV correspond to Co^3+^ and Co^2+^, respectively. The sub peaks at 796.65 eV and 794.07 eV are attributed to Co^2+^ and Co^3+^ in Co_2p1/2_, respectively. Therefore, we can confirm that the Co element in the sample exists in the form of Co^2+^ and Co^3+^. As Ce^3+^ replaces La^3+^, the relative concentration of Co^2+^ slightly increases. This is because when the doping of Ce^3+^ reaches saturation, some Ce^3+^ will transform into Ce^4+^ species. In order to maintain electronic neutrality, the valence state of some cobalt in the B site will change from Co^3+^ to Co^2+^. The increase in Co^2+^ content helps promote the formation of oxygen vacancies. Meanwhile, the presence of Ce also promotes the conversion of Co^2+^/Co^3+^ redox pairs, effectively improving the catalytic performance of the catalyst.

In Fig. 5c, the XPS spectrum of element La was successfully fitted as two 3d_3/2_ peaks (located at 849.79 eV and 853.79 eV) and two 3d_5/2_ peaks (located at 833.09 eV and 836.95 eV). In addition, the O 1s spectrum shown in Fig. 5d reveals two main oxygen species at 530.79 eV and 528.33 eV, corresponding to chemisorbed oxygen and surface lattice oxygen, respectively.

#### 3.1.5 NH_3_-TPD

The acidity of the catalyst has a significant impact on the depolymerization and reforming of oxygen-containing groups in lignin. This study determined the acidity of La_*x*_Ce_1−*x*_CoO_3_ (*X* = 0.2, 0.4, 0.6, 0.8, 1) series catalysts using NH_3_-TPD technology. According to the different desorption temperatures of NH_3_, acidic sites can be subdivided into weakly acidic sites (100–200 °C), moderately strong acidic sites (200–400 °C), and strongly acidic sites (>400 °C).

**Fig. 6:**
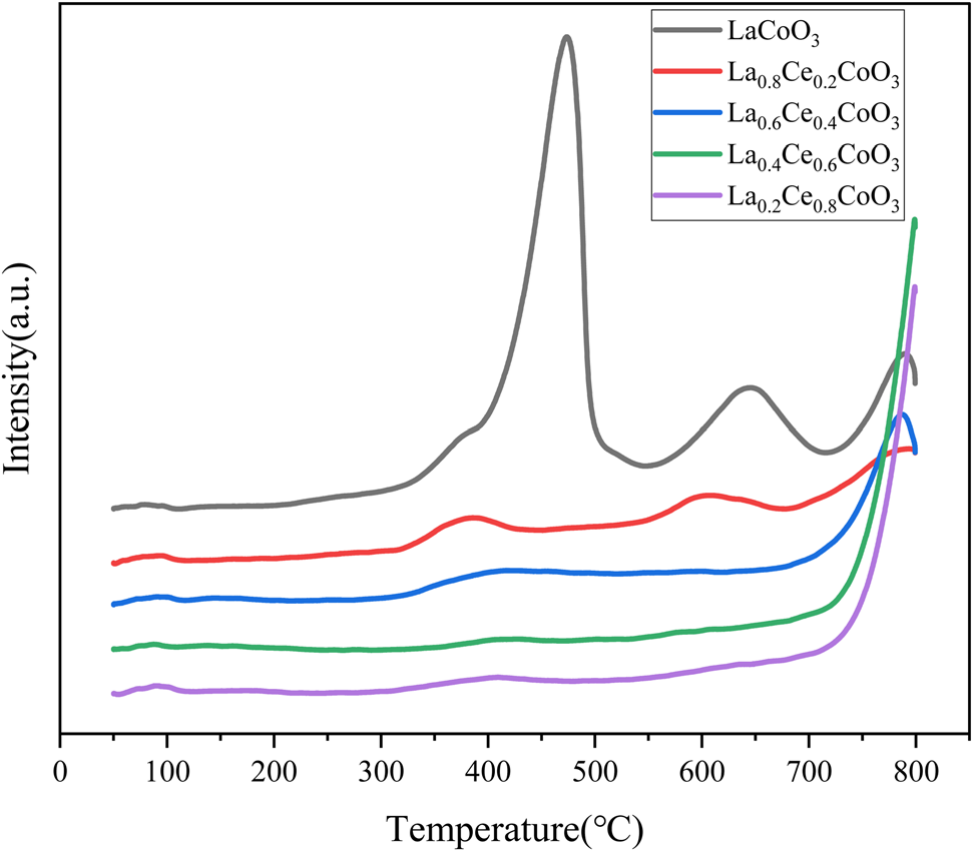
NH_3_-TPD curves of La_*x*_Ce_1−*x*_CoO_3_ (*x* = 0, 0.2, 0.4, 0.6, 0.8) series catalysts prepared.

As shown in Fig. 6, the LaCoO_3_ catalyst exhibits broad NH_3_ desorption peaks at 473.3 °C, 645.0 °C, and 789.8 °C, indicating that the catalyst only contains strong acidic sites. After doping with Ce element, the NH_3_ adsorption peaks of La_0.2_Ce_0.8_CoO_3_ and La_0.4_Ce_0.6_CoO_3_ are single, which may indicate that the high proportion of Ce element doping changes the original distribution of acidic sites, resulting in the disappearance or merging of some acidic sites into one main acidic site.

As the Ce doping ratio decreases, the strong acidic sites NH_3_ desorption peak of La_0.8_Ce_0.2_CoO_3_ and La_0.6_Ce_0.4_CoO_3_ catalysts gradually flattens, and more acidic sites of different intensities appear. This change indicates that moderate doping of Ce can increase the number of strong acidic sites in the catalyst, thereby enhancing its total acidity. This enhanced acidic site is beneficial for the activation and hydrogenolysis of C–O bonds by the catalyst, thereby improving the catalytic hydrogenolysis performance of La_0.6_Ce_0.4_CoO_3_.

### 3.2 Products yield

#### 3.2.1 The effects of different Ce doping levels

**Fig. 7:**
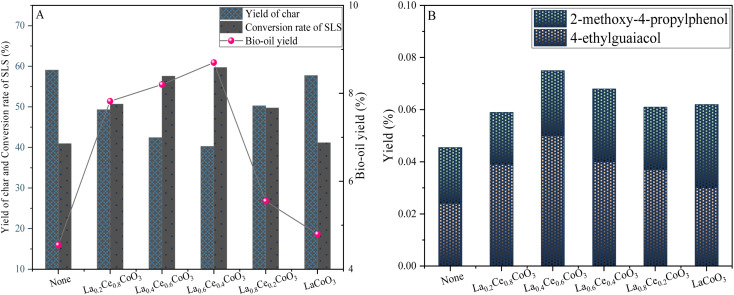
Yield plots of lignin sulfonate sodium depolymerization products (A) and phenolic products (B) for La_*x*_Ce_1−*x*_CoO_3_ (*x* = 0, 0.2, 0.4, 0.6, 0.8) series catalysts (0.6 g sodium lignosulfonate, 0.3 g catalyst, 240 °C, 1.1 MPa_N_2__, 12 h, 10 mL anhydrous ethanol and 10 mL isopropanol solvent system).

Fig. 7 illustrates the effect of different ratios of La_*x*_Ce_1−*x*_CoO_3_ (*x* = 0.2, 0.4, 0.6, 0.8, 1) catalysts on the depolymerization of lignin. In the absence of catalyst, the conversion rate of sodium lignosulfonate was only 40.96%, the yield of biooil was only 4.55 wt%, and most of the lignin was converted to coke (59.03 wt%), indicating that sodium lignosulfonate was not effectively depolymerized.

It is worth noting that all catalysts have some degree of improved product yield compared to non-catalytic experiments. With the increase of Ce doping from 0 to 0.4, the bio-oil yield increased significantly from 4.8 wt% to 8.7 wt%, and the yields of 4-ethylguaiacol and 2-methoxy-4-propylphenol were also significantly improved. At the same time, the conversion rate of lignin also increased from 41.16 wt% to 59.7 wt%. This increase may be attributed to the fact that the doping of Ce elements changed the crystal structure and surface properties of the LaCoO_3_ catalyst. We found that the specific surface area of the catalyst increased with the increase of Ce doping. For example, the specific surface area of the La_0.4_Ce_0.6_CoO_3_ catalyst is 6.5720 m^2^ g^−1^, which is increased compared to the undoped LaCoO_3_ (6.2792 m^2^ g^−1^), which is conducive to providing more active sites for the dispersion of the catalyst and the conversion of lignin (Table 1). However, the La_0.8_Ce_0.2_CoO_3_ catalyst has the largest specific surface area, but its depolymerization effect on lignin is not outstanding due to the low amount of Ce doping, relatively few active sites in the catalyst, and relatively limited redox ability. These results indicate that the change of Ce doping content is a key variable, but the effect on lignin catalytic depolymerization is nonlinear.

When the doping amount of Ce was increased to 0.4 to form La_0.6_Ce_0.4_CoO_3_ catalyst, although the specific surface area was the lowest among the La_*x*_Ce_1−*x*_CoO_3_ catalysts (*x* = 0.2, 0.4, 0.8, 1), the lignin conversion rate and bio-oil yield were the largest, indicating that although the specific surface area was an important factor affecting the catalytic effect in the La_*x*_Ce_1−*x*_CoO_3_ catalysts, it was not the only determinant. The activity of the catalyst is also affected by a combination of various factors such as acidity. Therefore, the reason why the La_0.6_Ce_0.4_CoO_3_ catalyst exhibits the best catalytic effect is that it achieves a relatively optimal equilibrium state in these aspects.

However, when the adulteration of Ce was further increased from 0.4 to 0.8, the yield of bio-oil decreased, from 8.7 wt% to 7.82 wt%, while the conversion rate of lignin also decreased from 59.7 wt% to 50.7 wt%. This phenomenon indicates that although the doping of too high Ce metal in La_0.4_Ce_0.6_CoO_3_ and La_0.2_Ce_0.8_CoO_3_ catalysts increases the active metal site, it also brings problems such as uneven metal distribution on the surface of the catalyst and excessive metal particle size, which may block the reaction site and lead to excessive hydrolysis of lignin.

Based on the above analysis, this study concluded that it is more appropriate to fix the doping amount of Ce metal in the catalyst at 0.6, which can not only ensure sufficient active sites, but also avoid the negative effects caused by excessive doping.

#### 3.2.2 Effects of reaction temperature

**Fig. 8:**
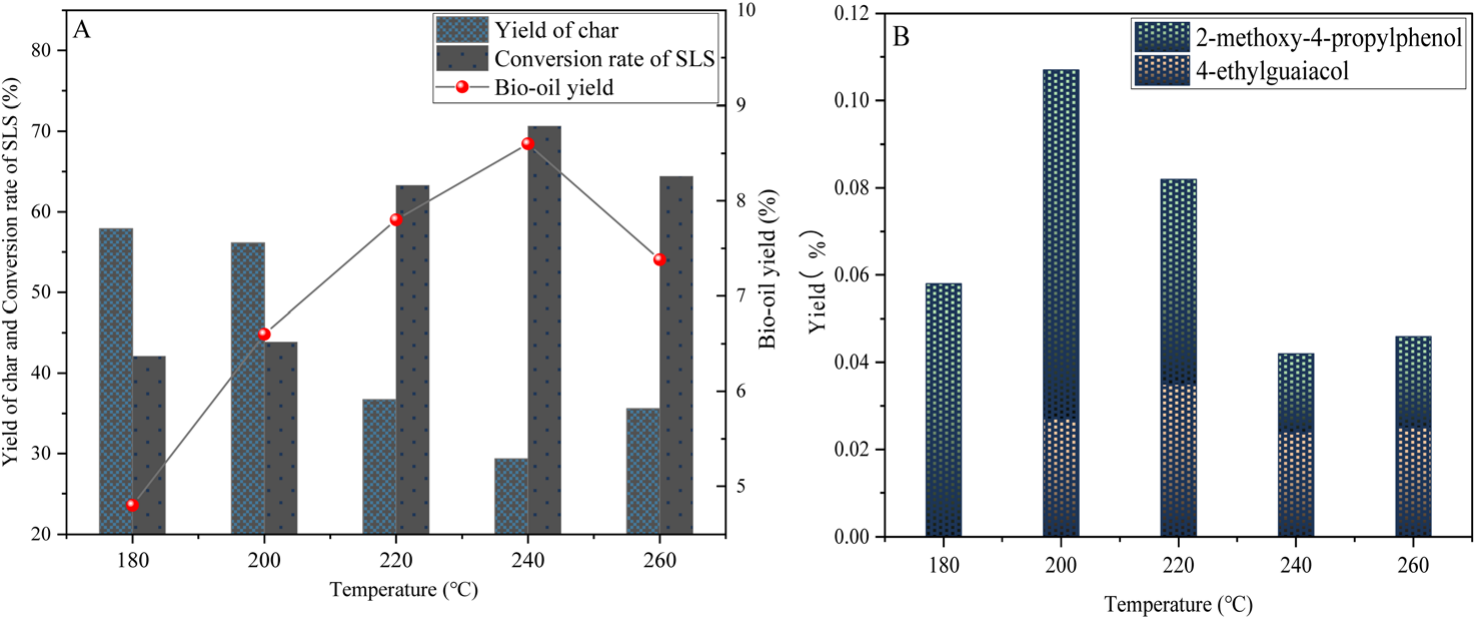
Yield of sodium lignosulfonate depolymerization products under temperature (A) and phenolic product yield (B) (0.6 g sodium lignosulfonate, 0.3 g catalyst, 1.1 MPa_N_2__, 12 h, 10 mL anhydrous ethanol and 10 mL isopropanol solvent system).

Fig. 8 reveals the changes in the depolymerization effect of lignin at different temperatures (180–260 °C), showing that with the temperature increasing from 180 °C to 240 °C, the bio-oil yield increases from 4.8 wt% to 8.6 wt%, the yield of 4-ethylguaiac is significantly increased, and the lignin conversion rate increases from 42.1 wt% to 70.65 wt%, but the bio-oil yield decreases to 7.38 wt% and the coke yield increases to 57.88 wt when the temperature is further increased to 260 °C.

This study indicates that when at the initial temperature point (180 °C), the vibration inside the lignin molecule is slowed down, which makes it difficult for the otherwise stable chemical bonds (such as β-O-4 ether bonds, α-O-4 ether bonds, *etc.*) to obtain enough energy to break. Therefore, the depolymerization effect of lignin under low temperature conditions is not good, and it is difficult to effectively release small molecule lignin fragments, which limits the formation of important phenolic compounds (such as 4-ethylguaiacol in bio-oil) in the subsequent conversion process.

However, with the gradual increase of temperature (200–240 °C), the depolymerization effect of lignin showed significant changes. This change has a profound impact on the production efficiency of bio-oil and the amount of carbon by-products generated.

When the temperature rises to 200 °C, the vibration of lignin molecules begins to intensify, making it easier for previously stable chemical bonds (such as β-O-4, α-O-4 ether bonds, *etc.*) to break, releasing more active intermediates. These intermediates are effectively adsorbed under the action of mesoporous catalysts, thereby limiting their free movement, reducing the chance of repolymerization, increasing their contact frequency with the active metal sites of the catalyst, promoting bio-oil yield and lignin conversion, which peaks at 240 °C.

At 240 °C, this effect reaches its peak, at which point the bio-oil yield and lignin conversion rate both reach their highest points within their respective temperature ranges. At the same time, the yield of phenolic substances such as 4-ethylguaiacol also significantly increases. However, it is worth noting that when the temperature further increased to 260 °C, the situation reversed. Although higher temperatures should theoretically be more conducive to the breaking of chemical bonds, at this time, the intermediate reaction activity is too strong, irregular collisions between molecules intensify, and condensation is prone to occur, ultimately forming a large amount of coke. Therefore, the yield of bio-oil decreased to 7.38 wt%, while the yield of coke sharply increased to 35.58 wt%.

Based on the study of temperature in the lignin depolymerization reaction and energy-saving considerations, 240 °C was identified as the ideal reaction temperature, and the yields of phenolic substances such as 4-ethylguaiacol and 2-methoxy-4-propyllignin have been significantly improved in this temperature range.

#### 3.2.3 Effects of reaction time

**Fig. 9:**
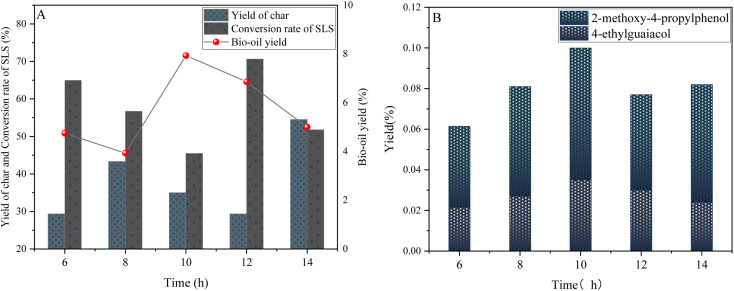
Time dependent graph of lignin sulfonate sodium depolymerization product yield (A) and phenolic product yield graph (B) (0.6 g sodium lignosulfonate, 0.3 g catalyst, 1.1 MPa_N_2__, 240 °C, 10 mL anhydrous ethanol and 10 mL isopropanol solvent system).

In the process of examining the catalytic depolymerization effect of lignin, reaction time was confirmed to be a crucial factor. As shown in Fig. 9(A), when the reaction time is increased from 6 hours to 10 hours, the bio-oil yield increases from 6.05 wt% to 8.26 wt%, which may result in the cleavage of more chemical bonds (β-O-4 ether bonds) in the lignin molecule, releasing more small molecule fragments. At the same time, the lignin conversion rate also increased from 51.78 wt% to 64.96 wt%, indicating that the extension of the reaction time promoted sufficient contact between the catalyst and the lignin molecule and improved the catalytic efficiency. In addition, the coke yield decreased from 44.8 wt% to 35 wt%, indicating that the priority of depolymerization reaction was enhanced and the condensation reaction was effectively inhibited within the appropriate reaction time.

However, when the reaction time was further extended to 12 hours, although the lignin conversion rate continued to increase to 70.65 wt%, the bio-oil yield unexpectedly decreased to 6.86 wt%, this phenomenon may be due to the inactivation of the active site as the reaction continued, and the active site was covered by the depolymerization product.

The effective specific surface area decreases and the catalytic efficiency decreases. At the same time, the coke yield also decreased slightly to 29.3 wt%, but this does not mean that all the depolymerization products are effectively converted to bio-oil, but some of the products may have formed more complex coke structures through condensation reactions.

The data in Fig. 9(B) confirms this trend: during the process of extending the reaction time from 6 hours to 10 hours, the yield of 4-ethylguaiacol significantly increases, indicating that the catalyst has a high selective catalytic ability for the cleavage of specific chemical bonds (such as ethyl and methoxy groups on the aromatic ring) within this time range. However, the yields of 4-ethylguaiacol and 2-methoxy-4-propylphenol decreased slightly at 12 and 14 hours, which may be related to changes in acidity. Excessive reaction time may lead to uneven acidity distribution on the surface of the catalyst, weaken its catalytic effect on the target phenolic compound, and at the same time promote the polycondensation reaction between the depolymerization products, thereby reducing the content of target phenols.

In summary, although prolonging the reaction time can improve the yield of mono-phenolic compounds within a certain range, too long reaction time will lead to polycondensation reaction of the depolymerization products due to factors such as excessive molecular cleavage, thermodynamic equilibrium shift, reduction of specific surface area and change of acidity, resulting in coke production and reduction of phenolic content. Therefore, considering the depolymerization effect and energy consumption, 10 hours was considered to be an ideal reaction time for the depolymerization effect of lignin.

#### 3.2.4 Effects of reaction solvent

Alcohol solvents play a key role in lignin-catalyzed hydrogenolysis. In order to test this hypothesis, we conducted a series of experiments to investigate the effects of different alcohol solvents and their mixtures on the hydrolysis effect of lignin.

**Fig. 10:**
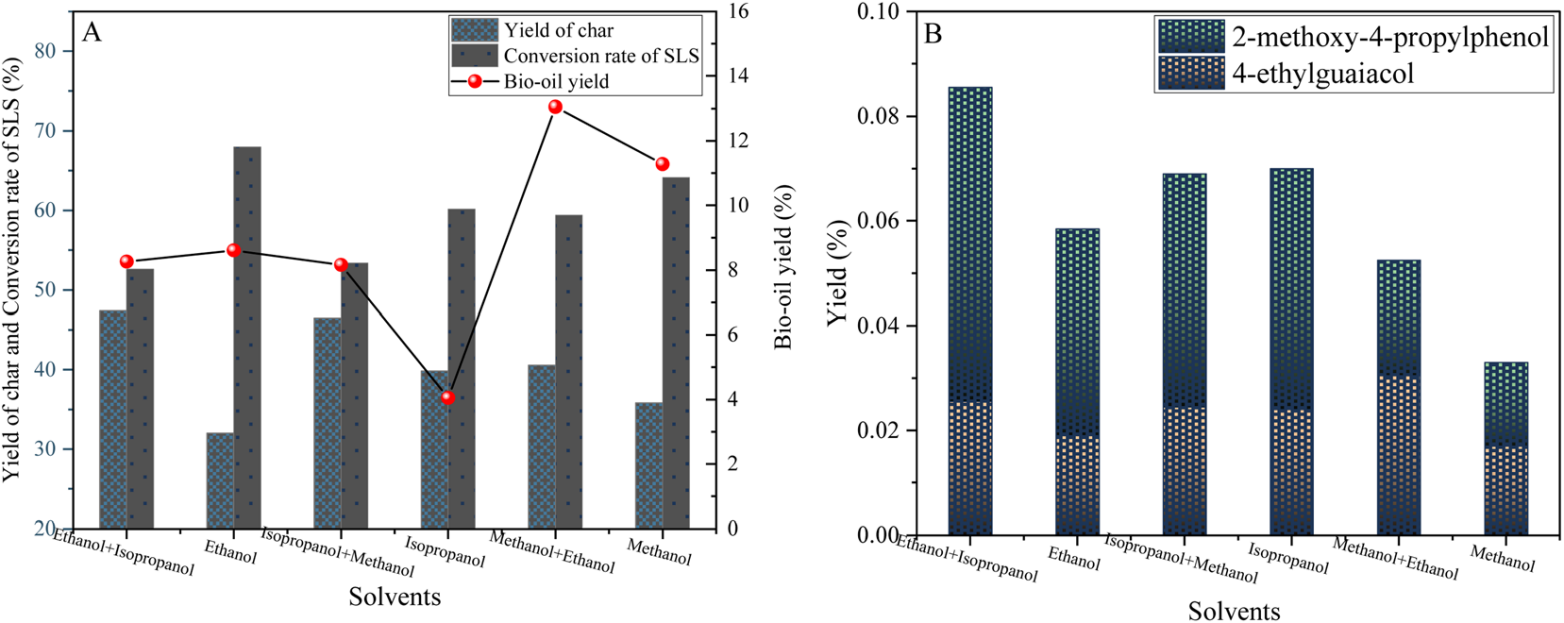
Yield plots of solvent induced depolymerization products of sodium lignosulfonate (A) and phenolic products (B) (0.6 g sodium lignosulfonate, 0.3 g catalyst, 1.1 MPa_N_2__, 10 h, 240 °C).

As shown in Fig. 10(A), when the mixed solvent of methanol and ethanol is used, the bio-oil yield is significantly increased to 13.05 wt%, which is much higher than that of single solvent methanol (8.61 wt%) or ethanol (11.28 wt%), and the carbon yield is also relatively low. This finding shows that the mixing of alcohol solvents as a good hydrogen donor can effectively enhance hydrogen transport and promote the hydrolysis of lignin.

Further analysis showed that the alcohol solvent not only served as the reaction medium, but also provided a stability mechanism for the lignin depolymerization process through its unique chemical properties. Specifically, the hydroxyl group of the alcohol solvent forms hydrogen bonds or other weak interactions with the active sites on the lignin fragment, stabilizing the depolymerized fragment that is susceptible to reaggregation or degradation (as described in Fig. 10(B)). This stabilization effect significantly reduces ineffective reaggregation reactions, increasing the purity and yield of the target product.

In particular, the blended solvents exhibit superior properties over the individual solvents. The mixing of alcohols such as methanol, ethanol and isopropanol optimizes the physicochemical properties of the solvent, such as solubility, polarity and hydrogen bond formation capacity, through the synergistic effect between the components. This optimization not only promotes the efficient transport of hydrogen, but also effectively inhibits the occurrence of adverse side reactions, so that the yield of target products such as 4-ethylguaiacol and 2-methoxy-4-propyllignanl is much higher than that of other solvent systems.

In conclusion, the alcohol solvents and their hybrid systems significantly improved the yield of biooil and the selectivity of target products by providing a stable chemical environment, promoting the transfer and utilization of hydrogen, and inhibiting the repolymerization of depolymerization products during lignin-catalyzed hydrogenolysis. This discovery not only provides a new strategy for the efficient conversion of lignin, but also opens up new avenues for the production of biofuels and chemicals.

#### 3.2.5 Comparison of catalytic effects of catalysts

The La_0.6_Ce_0.4_CoO_3_ perovskite catalyst developed in this study exhibits remarkable performance characteristics under specific reaction conditions (240 °C, 1.1 MPa N_2_).

Compared with ref. 28 and 29 its production process is simpler and does not require complex loading processes. Only a single catalyst is needed to achieve efficient catalysis, and it exhibits unique catalytic advantages under similar lignin depolymerization conditions.

The yield of its EA soluble product is as high as 13.05 wt%, which is at a relatively high level among similar catalysts (ref. 28 and 30), fully demonstrating the excellent catalytic activity of the catalyst. What is particularly outstanding is that the catalyst has excellent selectivity for the main product 4-ethylguaiacol, which has high industrial application value. In addition, the catalytic system can achieve efficient conversion under relatively mild reaction conditions (lower temperature and pressure), which not only reduces energy consumption but also provides convenience for subsequent industrial scaling up. In summary, the La_0.6_Ce_0.4_CoO_3_ catalyst has significant advantages in yield, product selectivity, and reaction conditions, making it an efficient and economical mild condition catalyst. In the future, by further optimizing reaction time and exploring more suitable reaction systems, its comprehensive performance is expected to be further improved (Table 2).

**Table 2:** Comparison of the catalytic effects of different catalysts on lignin depolymerization

Material	Catalytic	Reaction condition	Product	EA soluble product yield (wt%)	References
Cork lignin	V–Cu/ZrO_2_	150 °C, 5MPa_O_2__, 10 min	Vanillin	9	30
Organic solvent lignin	5%Pt–1%Ni/HTC	200 °C, 1.5 h, 2.0 MPa_H_2__	Phenol, 2-methoxyphenol	18	28
Sodium lignosulfonate	La_0.6_Ce_0.4_CoO_3_	240 °C, 10 h, 1.1 MPa_N_2__	4-Ethyl guaiacol	13.05	This chapter's work
Lignin	CoO/m-SEP	260 °C, 4 h, 4.0 MPa_H_2__	Phenol	8.12	31
Alkali lignin	10Ni10Fe/MgSiO_3_	300 °C, 1 h, 1.0 MPa_N_2__	Catechins, guaiacol, phenol	14.29	29

#### 3.2.6 Cyclic testing of catalysts

**Fig. 11:**
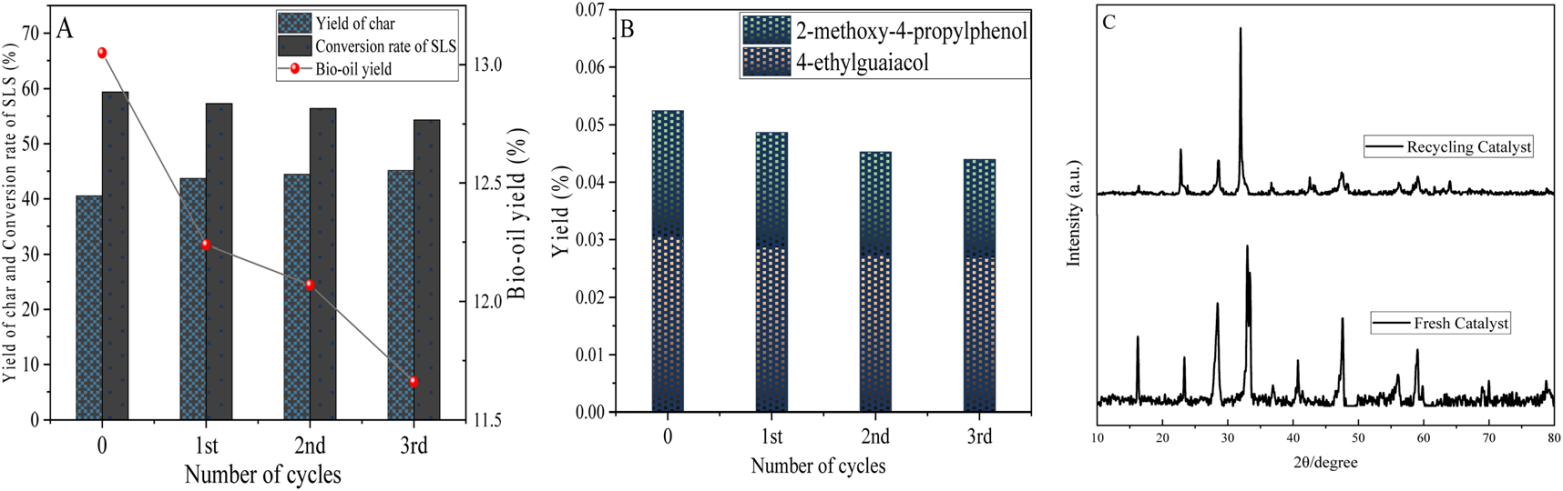
Test of catalytic depolymerization of lignin using recycled catalyst (A), phenolic product yield (B) and XRD analysis (C) (0.6 g sodium lignosulfonate, 0.3 g La_0.6_Ce_0.4_CoO_3_, 1.1 MPa_N_2__, 10 h, 240 °C, 10 mL methanol, 10 mL ethanol).

The stability and recyclability of catalysts are important reference variables for industrial applications. Under the same reaction conditions, three repeated experiments were conducted on the catalyst to study the catalytic effect of the regenerated catalyst. The results are shown in Fig. 11(A). As the number of cycles increases, the catalytic effect of La_0.6_Ce_0.4_CoO_3_ catalyst gradually weakens, which is reflected in the change of bio-oil yield (from 13.05 wt% to 11.66 wt%) and the increase of coke (from 40.56 wt% to 45.15 wt%). This may be due to the fact that the coke generated by the reaction not only directly physically covers some of the active sites, but also changes the microstructure of the catalyst surface by constructing complex carbonaceous deposits, thereby reducing the accessibility of the active sites and decreasing their effective utilization rate. Afterwards, a quantitative analysis was conducted on the yield of phenolic compounds, as shown in Fig. 11(B). It was found that after three cycles, the yields of 4-ethylguaiacol and 2-methoxy-4-propylphenol were relatively stable and did not significantly decrease. This indicates that the catalyst still has high selectivity and stability towards the target phenolic compound with increasing circulation.

Fig. 11(C) shows the XRD patterns of fresh catalyst and old catalyst recovered after 3 cycles. It can be seen that the characteristic peak intensity of CeO_2_ is significantly reduced at 2*θ* = 28.5°, 32.9°, and 47.5°, while only an extremely weak characteristic peak of La_2_O is detected at 2*θ* = 58.92°, indicating that the metal elements on the catalyst surface have partially detached during multiple lignin depolymerization processes, causing changes in its crystal structure, resulting in surface roughness, element loss, and limiting the diffusion and adsorption ability of reactant molecules on the catalyst surface.

Despite these unfavorable factors, the results of the third cycle experiment (lignin conversion rate: 54.3 wt%, product oil yield: 11.66 wt%) were still far better than those of the experiment without catalyst (lignin conversion rate: 40.96 wt%, product oil yield: 4.55 wt%). After 3 cycles, the catalyst still maintains a certain level of catalytic activity. Therefore, the La_0.6_Ce_0.4_CoO_3_ catalyst exhibits good stability and recyclability in the process of lignin catalyzed depolymerization.

### 3.3 Characterization of bio-oils

#### 3.3.1 Gas chromatography/mass spectroscopy (GC/MS) analysis

**Fig. 12:**
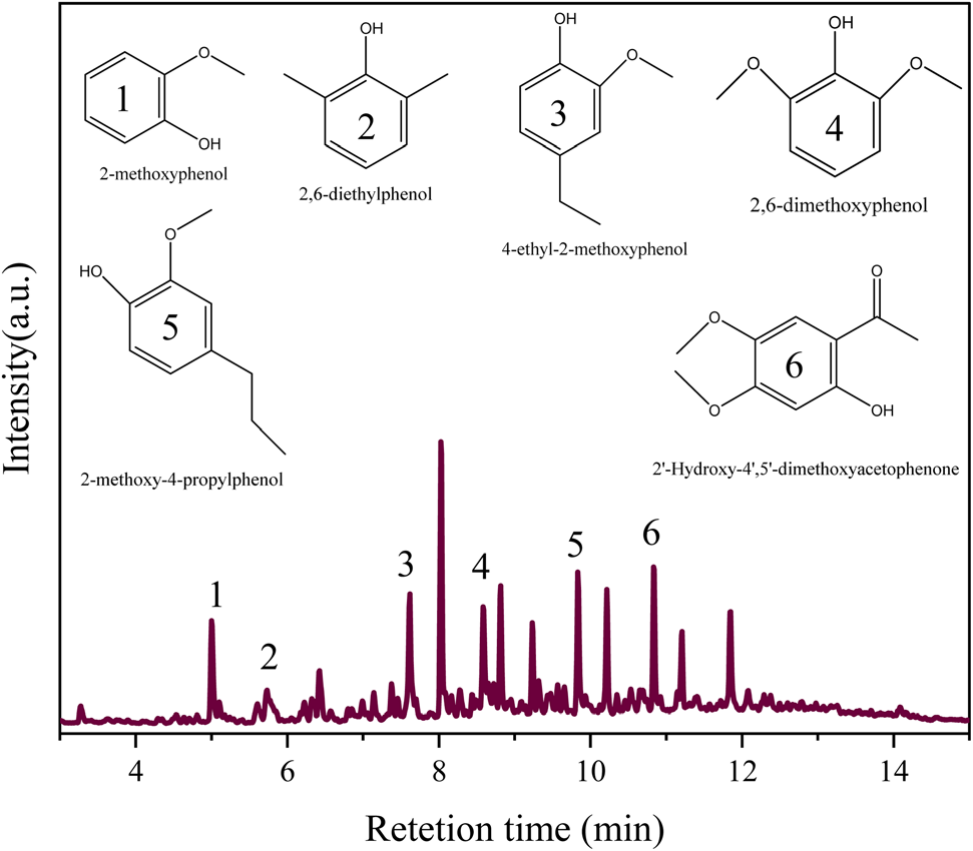
GC-MS analysis of the depolymerization products of sodium lignosulfonate by La_0.6_Ce_0.4_CoO_3_ (0.6 g sodium lignosulfonate, 0.3 g catalyst, 1.1 MPa_N_2__, 240 °C, 10 h, 10 mL ethanol and 10 mL methanol solvent system).

Fig. 12 shows the product distribution of monomer compounds in bio-oil obtained by La_0.6_Ce_0.4_CoO_3_ catalyst under optimal reaction conditions. Except for the peak caused by the solvent ethyl acetate and its derivatives, the main depolymerization products of lignin are guaiacol, 2,6-dimethylphenol, 4-ethylguaiacol, 2,6-dimethoxyphenol, and 2-methoxy-4-propylphenol. Among them, phenols with ethyl and ester groups may be formed by secondary reactions such as alkylation and esterification between phenolic intermediates and ethanol. Under supercritical conditions, the O-alkylation and *C*-alkylation reactions of phenol with alcohols can occur spontaneously without the involvement of additional catalysts.

In addition to phenolic compounds, a small amount of fatty ester compounds such as ethyl dodecanoate are also generated during the depolymerization process, which may originate from triglyceride impurities in lignin raw materials. By optimizing the reaction conditions and using La_0.6_Ce_0.4_CoO_3_ catalyst for lignin depolymerization reaction, the total yield of monomer compounds was significantly improved, far higher than the yield under no catalyst conditions. This discovery not only demonstrates the high efficiency of La_0.6_Ce_0.4_CoO_3_ catalyst in lignin depolymerization reaction, but also opens up new avenues for high-value utilization of lignin. These phenolic compounds have broad application prospects in chemical, pharmaceutical and other fields, providing strong technical support for the deep development of lignin resources.

#### 3.3.2 FT-IR analysis of the bio-oils

**Fig. 13:**
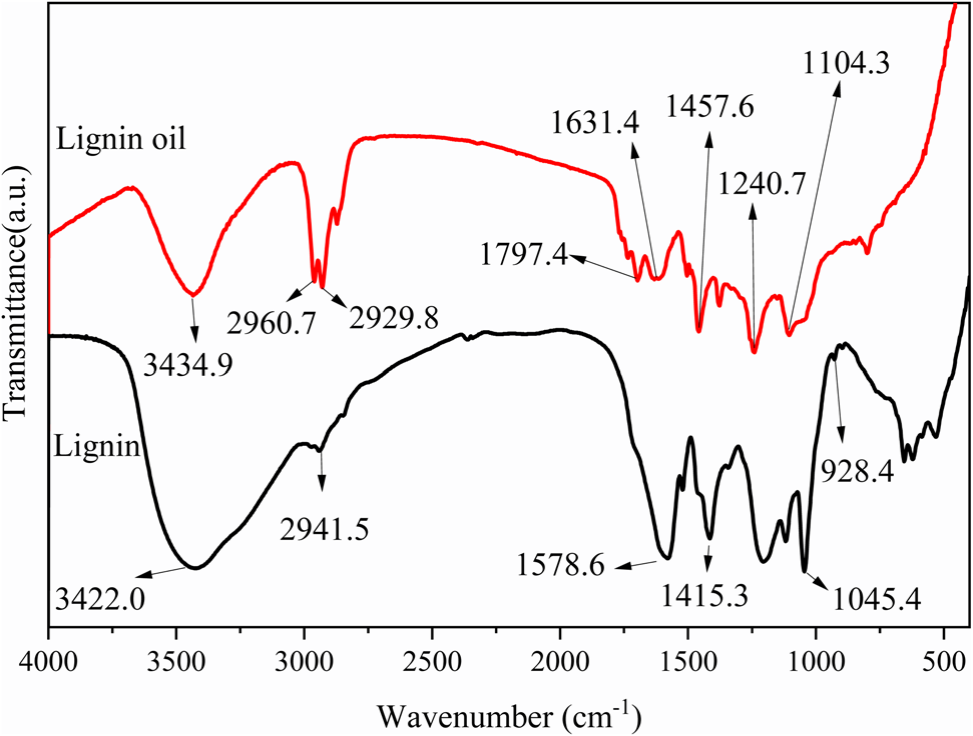
FT-IR spectra of sodium lignosulfonate and lignin oil.

Fig. 13 shows the FTIR spectra of lignin and bio-oil. The peak near 3440 cm^−1^ is caused by the stretching vibration of hydroxyl (–OH), while the peaks at 2939 cm^−1^ and 2856 cm^−1^ are caused by the stretching vibration of methyl (–CH_3_) and methylene (–CH_2_–). It is not difficult to see that after the reaction, the lignin structure has been destroyed. For primitive lignin, the absorption peaks at 2945 cm^−1^ (–CH_3_ and –CH_2_–) and 928 cm^−1^, 625 cm^−1^ disappear. For lignin oil, C–H stretching vibration of methylene group and C–O–C stretching vibration at 1116 cm^−1^ were observed at 2866 cm^−1^.

The appearance of these absorption peaks also indirectly confirms the structure of the polymer products and indicates that after catalytic action, lignin will be efficiently converted into guaiacol and its derivatives. In addition, stretching vibration peaks of other specific functional groups were also observed. For example, the absorption peaks appearing near 3400 cm^−1^, 1600 cm^−1^, 1514 cm^−1^, and 800 cm^−1^ are caused by the stretching vibrations of phenolic hydroxyl, aliphatic hydroxyl, and aromatic ring outer plane C–H.

The –OH vibration signal of bio-oil is significantly enhanced, and the peak range of the signal is the widest, indicating abundant hydrogen bonds within the bio-oil molecules. In addition, during the process of lignin depolymerization to generate bio-oil, although chemical bonds are broken and recombined, some major functional groups such as methyl, methoxy, hydroxyl, *etc.* still remain in the bio-oil. These functional groups are relatively stable during the pyrolysis process and are not easily completely destroyed or converted. In addition, the similarity between the infrared spectra of the two is high, which means that the extraction process of bio-oil is more effective and can better preserve the effective components in lignin.

#### 3.3.3 Elemental analysis of the bio-oils

Table 3 lists the C, H, O, N elemental analysis and calorific value (HHV) of lignin oil and native lignin generated by catalyst depolymerization under optimal reaction conditions. From the table, it can be seen that the C and H elements in the depolymerized product oil have increased to varying degrees, while the O element content has decreased. Compared with the high heating value (HHV) calculated by the Dulong formula, the catalyst corresponds to the highest calorific value of the product, increasing from 9.06 MJ kg^−1^ to 18.74 MJ kg^−1^, the H/C ratio has increased from 0.13 to 0.16, and the O/C ratio has decreased from 1.58 to 0.96. This indicates that lignin depolymerization involving catalysts has undergone an effective hydrogenation deoxygenation process.

**Table 3:** Analysis of elements and calorific value of lignin decomposition products

Sample	Element content, (wt%)							
	C	H	O	N	S	O/C	H/C	HHV (MJ kg^−1^)
Lignin	34.17	4.19	54.08	0.10	7.46	1.58	0.13	9.06
Lignin oil	45.56	7.24	44.1	0.19	2.91	0.96	0.16	18.74

#### 3.3.4 ^1^H NMR analysis of lignin oil

**Fig. 14:**
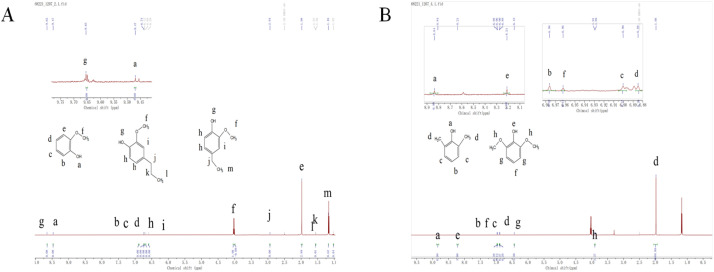
1H NMR spectra of La_0.6_Ce_0.4_CoO_3_ depolymerized bio-oil (A) and none catalyst bio-oil 1H NMR spectra (B) (0.6 g sodium lignosulfonate, 1.1 MPa_N_2__, 240 °C, 10 h, 10 mL ethanol and 10 mL methanol solvent system).

Presence of phenolic structures, but their signal intensity is significantly weakened compared to Fig. 14(A). In addition, within the characteristic region (9.0–10.0 ppm), Fig. 14(A) exhibits a sharp single peak at 9.5–9.7 ppm, representing the active hydrogen in the phenolic hydroxyl group, while Fig. 14(B) shows a signal around 8.8 ppm, but the difference in chemical shift indicates that the substitution position of the phenolic hydroxyl group or the electronic effect of surrounding groups in its molecular structure is different.

Based on the above results, the sample in Fig. 14(A) is rich in phenolic hydroxyl groups and methylated phenolic compounds, and also has many oxidation sites, which is consistent with the results of FTIR and GC-MS.

In contrast, the phenol content in Fig. 14(B) is lower, and the aromatic hydrogen signal is weakened, which may be attributed to the higher degree of methoxy substitution, leading to an enhanced shielding effect of the aromatic ring. These differences reflect that the catalyst (La_0.6_Ce_0.4_CoO_3_) is more effective in depolymerizing lignin.

### 3.4 Lignin depolymerization mechanism

GC-MS analysis results based on EA soluble products and relevant literature reports. We have made a reasonable speculation on the depolymerization mechanism of sodium lignosulfonate (SLS) under the action of perovskite oxide catalyst. Perovskite oxide catalysts have the characteristic of releasing adsorbed oxygen and lattice oxygen at high temperatures, and supplementing lattice oxygen during the regeneration process of perovskite.^32^ During the pyrolysis of lignin, the adsorbed oxygen and lattice oxygen released by perovskite at high temperatures oxidize Cα–OH in SLS to Cα=O, thereby reducing the bond energy of Cα–Cβ bonds, lowering the energy threshold for bond cleavage, and promoting the decomposition of lignin structure.^33,34^ During this process, alcohols (methanol and ethanol) play a crucial role as *in situ* hydrogen donors. They generate highly reactive free radicals such as hydrogen atoms, methoxy groups (–OCH_3_), and ethoxy groups (–OC_2_H_5_) through decomposition reactions.^35^ These free radicals not only promote the catalytic hydrogenation process, but also selectively act on the C–O bonds of lignin aryl ether adsorbed and activated by oxygen vacancies in perovskite catalysts, especially the β-O-4 and α-O-4 bonds, leading to effective cleavage of these chemical bonds. As the depolymerization reaction progresses, these free radicals generated by alcohol decomposition undergo structural rearrangement under the combined influence of catalytic activity at the active sites of the catalyst and thermal effects, further promoting the cleavage of Cα–Cβ bonds. This series of reactions ultimately formed highly reactive phenolic intermediates such as 3,5-diethyl-4-hydroxyacetophenone and ferulic acid, providing an important foundation for subsequent conversion and utilization. In addition, ethanol can stabilize phenolic intermediates through *O*-alkylation and *C*-alkylation, effectively inhibit the re polymerization reaction, avoid the formation of carbon residue, and improve monomer yield.^36^ Subsequently, we emphasized the important role of catalysts in the further cracking process of lignin.

The valence state cycling of Ce^4+^/Ce^3+^ of cerium (Ce) element in the catalyst (La_0.6_Ce_0.4_CoO_3_) promotes the formation of oxygen vacancies on the surface of CeO_2_, effectively inhibits coking during the catalytic process, improves the selectivity of target phenolic compounds, and further decomposes aromatic intermediates into mono-phenolic compounds such as cresol and 2,6-dimethylphenol.^37^

**Fig. 15:**
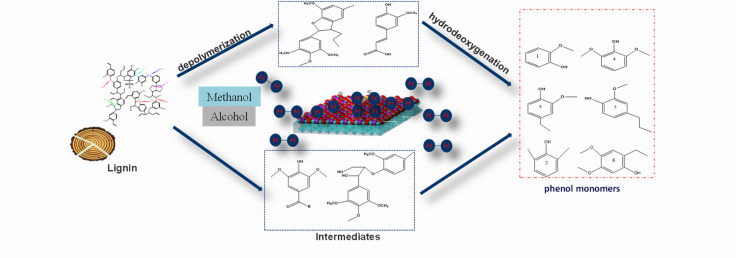
Possible pathways of SLS catalytic depolymerization to monomeric phenols.

Finally, we summarized the key role of the La_0.6_Ce_0.4_CoO_3_ catalyst in this reaction system. Its highly dispersed active sites promote the release of active hydrogen, while the abundant mesoporous structure and oxygen vacancies significantly improve the adsorption and activation performance of the catalyst for lignin molecular fragments. Together, these properties make the catalyst excellent for promoting lignin depolymerization, improving biooil yield, and high selectivity for specific compounds (Fig. 15).

### 3.5 DFT simulation calculation

**Fig. 16:**
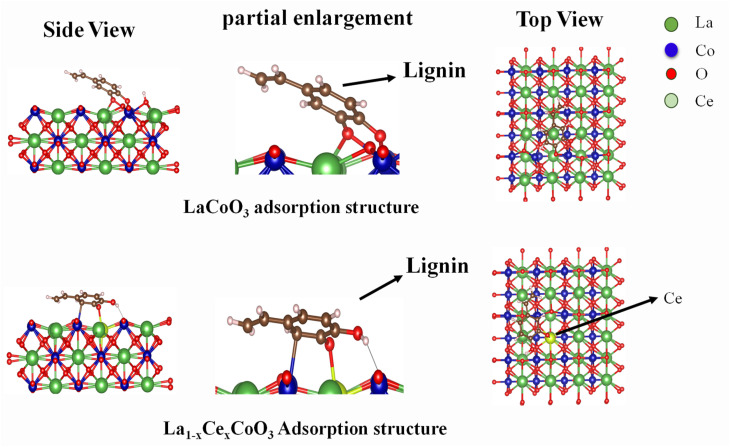
Side and top views of DFT calculation reaction model of lignin on La_*x*_Ce_1−*x*_ CoO_3_/LaCoO_3_.

In order to further elucidate the possible reaction process of lignin cracking catalyzed by La_0.6_Ce_0.4_CoO_3_, DFT simulation calculations were conducted on the hydrogenolysis reaction of lignin on La_0.6_Ce_0.4_CoO_3_. As shown in Fig. 16, all spin calculations were performed within the framework of density functional theory (DFT) using the projection enhanced plane wave method, as implemented in the Vienna *ab initio* simulation package (VASP).^38,39^ The general gradient approximation proposed by Perdew, Burke, and Ernzerhof was chosen as the Exchange Correlation Potential (GGA-PBE).^40,41^ The cutoff energy of plane waves is set to 400 eV. In the iterative solution of the Kohn Sham equation, the energy standard is set to 1 × 10^−6^ eV. The van der Waals interaction was incorporated into Grimme's DFT-D3 method.^42^ The (110) surface of LaCoO_3_ is represented as LaCoO_3_ catalyst, and the substitution of one La atom with Ce atom is represented as Ce doped LaCoO_3_ catalyst. Apply a vacuum thickness of 15 Å along the *z*-direction to avoid periodic interactions. A 2 × 2 × 1 Monkhorst Pack grid was used in K-sampling. A lignin molecular model was constructed and optimized in 15 × 15 × 15 Å cubic cells with a single *k*-point.

In the undoped LaCoO_3_ system, lignin molecules tend to adsorb onto the catalyst surface in a tilted manner through the O end, forming O–Co bonds with bond lengths of 0.214 nm and 0.206 nm, respectively. Although this adsorption mode exhibits relatively high adsorption energy, the large interaction angle between molecules and catalyst surfaces limits the effective contact area between lignin and catalyst, thereby affecting adsorption stability. In contrast, Ce doped LaCoO_3_ catalyst promotes parallel adsorption of lignin molecules with C and O atoms on the surface, forming C–Co bonds (0.216 nm) and O–Ce bonds (0.228 nm). This parallel adsorption mode significantly increases the contact area between molecules and catalysts, not only improving adsorption stability, but also reducing the total energy of the system, which is beneficial for lignin depolymerization reaction. The introduction of Ce adds additional active sites to the catalytic system, such as Ce–O bonds. Meanwhile, the variable valence state of Ce^3+^/Ce^4+^ and the formation of oxygen vacancies significantly improve the electronic conductivity and bond activation efficiency of the catalyst. In summary, the cerium doped LaCoO_3_ catalyst not only significantly reduces the activation energy of lignin depolymerization, but also exhibits excellent catalytic performance. This provides new research directions and technical support for the efficient conversion of lignin.

## 4 Conclusions

In the catalytic depolymerization of lignin to produce monophenols using La_*x*_Ce_1−*x*_CoO_3_ (*x* = 0, 0.2, 0.4, 0.6, 0.8) perovskite oxide catalysts prepared by co precipitation method, the lignin conversion rate and carbon yield are closely related to the doping amount of metal Ce. Under the optimal reaction conditions, the catalytic conversion rate of lignin can reach 59.4%. Compare with non-catalytic processes, catalytic processes significantly improve the conversion rate of lignin and the yield of bio-oil. The results showed that the perovskite catalyst La_*x*_Ce_1−*x*_CoO_3_ (*x* = 0, 0.2, 0.4, 0.6, 0.8) also exhibited significant activity and structural stability in the lignin catalyzed depolymerization process, making it an efficient multiphase catalyst in lignin catalysis.

## Data availability

The data supporting this article can be found in the following websites (published papers). (1) https://www.sciencedirect.com/science/article/abs/pii/S0016236121029604 (2) https://www.nature.com/articles/s41929-017-0007-z (3) https://www.sciencedirect.com/science/article/pii/S2468025721000261 (4) https://pubs.acs.org/doi/10.1021/acssuschemeng.9b05041.

## Conflicts of interest

There are no conflicts to declare.

## References

[cit1] Yang Y. Q., Xiao L. P., Xiao W. Z., Li X. Y., Wang. Q., Sun R. C. (2022). Nitrogen-doped carbon anchored ruthenium nanoparticles for biofuel upgrade. Fuel.

[cit2] Sun Z. H., Bottari G., Afanasenko A., Marc C. A. S., Peter J., Katalin B. (2018). Complete lignocellulose conversion with integrated catalyst recycling yielding valuable aromatics and fuels. Nat. Catal..

[cit3] Ciaran W. L., Kamer P. C. J., Lancefield C. S., Deuss P. J. (2020). An Introduction to Model Compounds of Lignin Linking Motifs; Synthesis and Selection Considerations for Reactivity Studies. ChemSusChem.

[cit4] Zhang C. F., Wang F. (2020). Catalytic Lignin Depolymerization to Aromatic Chemicals. Acc. Chem. Res..

[cit5] Schutyser W., Renders T., Van den Bosch S., Koelewijn S. F., Beckham G. T., Sels B. F. (2018). Chemicals from lignin: an interplay of lignocellulose fractionation Depolymerization, and upgrading. *Chem. Soc. Rev*
**.**.

[cit6] Fan Y. Y., Liu C., Kong X. C., Han Y., Lei M., Xiao R. (2021). A new perspective on polyethylene-promoted lignin pyrolysis with mass transfer and radical explanation. Green Energy Environ..

[cit7] Chen S. S., Lu Q. Q., Han W. Y., Yan P. X., Wang H. L., Zhu W. B. (2021). Insights into the oxidation–reduction strategy for lignin conversion to high-value aromatics. Fuel.

[cit8] Biswas B., Kumar A., Krishna B. B., Bhaskar T. (2021). Effects of solid base catalysts on depolymerization of alkali lignin for the production of phenolic monomer compounds. Renewable Energy.

[cit9] Liu X. D., Jiang Z. C., Feng S. S., Z H., Li J. M., Hu C. W. (2019). Catalytic depolymerization of organosolv lignin to phenolic monomers and low molecular weight oligomers. Fuel.

[cit10] Barta K., Matson T. D., Fettig M. L., Scott S. L., Iretskii A. V., Ford P. C. (2010). Catalytic disassembly of an organosolv lignin via hydrogen transfer from supercritical methanol. Green Chem..

[cit11] Ma H. W., L H. W., Zhao W. J., Li L. X., Liu S. J., Long J. X., Li X. H. (2019). Selective depolymerization of lignin catalyzed by nickel supported on zirconium phosphate. Green Chem..

[cit12] Shu R. Y., Li R. X., Lin B. Q., Luo B. W., Tian Z. P. (2020). High dispersed Ru/SiO_2_-ZrO_2_ catalyst prepared by polyol reduction method and its catalytic applications in the hydrodeoxygenation of phenolic compounds and pyrolysis lignin-oil. Fuel.

[cit13] Zhu. C., Ding S. Y., Hojo H., Einaga H. (2021). Controlling Diphenyl Ether Hydrogenolysis Selectivity by Tuning the Pt Support and H-Donors under Mild Conditions. ACS Catal..

[cit14] Álvarez B. P., Martin R. (2010). Ni-Catalyzed Reduction of Inert C−O Bonds: A New Strategy for Using Aryl Ethers as Easily Removable Directing Groups. J. Am. Chem. Soc..

[cit15] Qi G. S., Joseph E., Ralph T. (2004). Selective Catalytic Reduction of NO with NH_3_ over Fe-ZSM-5 Catalysts Prepared by Sublimation of FeCl_3_ at Different Temperatures. Catal. Lett..

[cit16] Jeong S., Yang S., Kim D. H. (2017). Depolymerization of Protobind lignin to produce monoaromatic compounds over Cu/ZSM-5 catalyst in supercritical ethanol. Mol. Catal..

[cit17] Hao G. G., Liu H. Y., Chang Z. B., Song K. C., Yang. X., Ma H., Wang W. G. (2022). Catalytic depolymerization of the dealkaline lignin over Co–Mo–S catalysts in supercritical ethanol. Biomass Bioenergy.

[cit18] Zhao W. G., Li X., Li H. W., Zheng X. L., Ma H. W., Long G. X., Li X. H. (2019). Selective Hydrogenolysis of Lignin Catalyzed by the Cost-Effective Ni Metal Supported on Alkaline MgO. ACS Sustainable Chem. Eng..

[cit19] Jiang B. X., Hu G., Qiao Y. H., Jiang X. X., Lu P. (2019). Depolymerization of lignin over Ni-Pd bimetallic catalyst using isopropanol as in situ hydrogen source. Energy Fuels.

[cit20] Todorov T., Gunawan O., Jay Chey S., Mitzi D. B. (2011). Progress towards applications of perovskite solar cells. Thin Solid Films.

[cit21] Shellaiah M., Sun K. W. (2020). Review on Sensing Applications of Perovskite Nanomaterials. Chemosensors.

[cit22] Sun. N., Jin F. G., Liu X. L., Liu X. W., Li G. X., Shen Y., Wang F., Chu X. Y., Wu Z., Li G. H., Lv X. L. (2021). In Situ Co-exsolution of Metal Nanoparticle-Decorated Double Perovskites as Anode Materials for Solid Oxide Fuel Cells. ACS Appl. Energy Mater..

[cit23] Deng H. B., Lin. L., Sun Y., Pang C. S., Zhuang J. P., Ouyang P. K., Li J. J., Liu S. J. (2009). Activity and Stability of Perovskite-Type Oxide LaCoO_3_ Catalyst in Lignin Catalytic Wet Oxidation to Aromatic Aldehydes Process. Energy Fuels.

[cit24] Deng H. B., Lin. L., Sun. Y., Pang C. S., Zhuang J. P., Ouyang P. K., Li Z. J., Liu S. J. (2008). Perovskite-type Oxide LaMnO_3_: An Efficient and Recyclable Heterogeneous Catalyst for the Wet Aerobic Oxidation of Lignin to Aromatic Aldehydes. Catal. Lett..

[cit25] Zhang J. H., Deng H. B., Lin L. (2009). Wet Aerobic Oxidation of Lignin into Aromatic Aldehydes Catalysed by a Perovskite-type Oxide: LaFe_1-x_Cu_x_O_3_ (x=0, 0.1, 0.2). Molecules.

[cit26] Deng H. B., Lin L., Liu S. J. (2010). Catalysis of Cu-Doped Co-Based Perovskite-Type Oxide in Wet Oxidation of Lignin To Produce Aromatic Aldehydes. Energy Fuels.

[cit27] Li Y. X., Zhu J. P., Zhang Z. J., Qu Y. S. (2020). Preparation of Syringaldehyde from Lignin by Catalytic Oxidation of Perovskite-Type Oxides. ACS Omega.

[cit28] Süss R., Aufischer G., Zeilerbauer L., Kamm B., Meissner G., Spod H., Paulik C. (2022). Depolymerization of organosolv lignin by supported Pt metal catalysts. Catal. Commun..

[cit29] Laobuthee A., Khankhuean A., Panith P., Veranitisagul C., Laosiripojana N. (2023). Ni–Fe Cocatalysts on Magnesium Silicate Supports for the Depolymerization of Kraft Lignin. ACS Omega.

[cit30] Abdelaziz O. Y., Clemmensen I., Meier S., Bjelić S., Hulteberg C. P., Riisager A. (2023). Oxidative Depolymerization of Kraft Lignin to Aromatics Over Bimetallic V–Cu/ZrO2 Catalysts. Top. Catal..

[cit31] Chen M. Q., Cao Y., Wang Y. S., Yang Z. L., Wang Q., Sun Q. Q., Wang J. (2019). Depolymerization of lignin over CoO/m-SEP catalyst under supercritical methanol. J. Renewable Sustainable Energy.

[cit32] Zhao K., He. F., Huang. Z., Wei G. Q., Zheng A. Q., Li H. B., Zhao Z. L. (2016). Perovskite-type oxides LaFe1−xCoxO3
for chemical looping steam methane reforming to syngas and hydrogen co-production. Appl. Energy.

[cit33] Borel L. D. M. S., Lira T. S., Ribeiro J. A., Ataíde C. H., Barrozo M. A. S. (2018). Pyrolysis of brewer's spent grain: Kinetic study and products identification. Ind. Crops Prod..

[cit34] Nair V., Vinu R. (2016). Production of guaiacols via catalytic fast pyrolysis of alkali lignin using titania, zirconia and ceria. J. Anal. Appl. Pyrolysis.

[cit35] Kleinert M., Gasson J. R., Barth T. (2009). Optimizing solvolysis conditions for integrated Depolymerization and hydrodeoxygenation of lignin to produce liquid biofuel. J. Anal. Appl. Pyrolysis.

[cit36] Huang X., Korányi T. I., Boot M. D., Hensen E. J. M. (2015). Catalytic Depolymerization of Lignin in Supercritical Ethanol. ChemSusChem.

[cit37] Lei L. J., Wang Y. H., Zhang Z. X., An J. H., Wang F. (2020). Transformations of Biomass, Its Derivatives, and Downstream Chemicals over Ceria Catalysts. ACS Catal..

[cit38] Kresse. G. G., Furthmüller J. J. (1996). Efficient Iterative Schemes for Ab Initio Total-Energy Calculations Using a Plane-Wave Basis Set. Phys. Rev. B:Condens. Matter Mater. Phys..

[cit39] Kresse a. G., Furthmüller b J. (1996). Efficiency of ab-initio total energy calculations for metals and semiconductors using a plane-wave basis set. Comput. Mater. Sci..

[cit40] Perdew. J. P., Burke. K., Ernzerhof M. (1996). Generalized Gradient Approximation Made Simple. Phys. Rev. Lett..

[cit41] White. J. A., Bird-Implementation D. M. (1994). of Gradient-Corrected Exchange-Correlation Potentials in Car-Parrinello Total-Energy-Calculations. Phys. Rev. B:Condens. Matter Mater. Phys..

[cit42] Antony. S. G. J., Ehrlich. S., Krieg H. (2010). A consistent and accurate ab initio parametrization of density functional dispersion correction (DFT-D) for the 94 elements H-Pu. J. Chem. Phys..

